# Single nucleotide polymorphisms in *ZNRD1-AS1* increase cancer risk in an Asian population

**DOI:** 10.18632/oncotarget.14334

**Published:** 2016-12-28

**Authors:** Ping-Yu Wang, Jing-Hua Li, Yue-Mei Liu, Qing Lv, Ning Xie, Han-Han Zhang, Shu-Yang Xie

**Affiliations:** ^1^ Key Laboratory of Tumor Molecular Biology in Binzhou Medical University, Department of Biochemistry and Molecular Biology, Binzhou Medical University, YanTai, ShanDong, 264003, P.R.China; ^2^ Department of Epidemiology, Binzhou Medical University, YanTai, ShanDong, 264003, P.R.China; ^3^ Department of Chest Surgery, YanTaiShan Hospital, YanTai, ShanDong, 264000, P.R.China

**Keywords:** ZNRD1-AS1, single nucleotide polymorphism, cancer, meta-analysis, lncRNA

## Abstract

Single nucleotide polymorphisms (SNPs) in human zinc ribbon domain containing 1 antisense RNA 1 (*ZNRD1-AS1*) have been associated with cancer development. In this meta-analysis, we more precisely estimated the associations between three expression quantitative trait loci SNPs in *ZNRD1-AS1* (rs3757328, rs6940552, and rs9261204) and cancer susceptibility. The data for three SNPs were extracted from eligible studies, which included 5,293 patients and 5,440 controls. Overall, no significant associations between SNPs in *ZNRD1-AS1* (rs3757328, rs6940552, and rs9261204) and cancer risk were observed. However, in further subgroup analyses based on cancer type, we found that the A allele of rs3757328 increased the risk of some cancer in both allele contrast (OR = 1.15, 95% CI = 1.05 – 1.25) and recessive models (OR = 1.79; 95% CI = 1.33 – 2.41). The A allele of rs6940552 and the G allele of rs9261204 also increased the risk of some cancer in an Asian population in allele contrast (OR = 1.17, 95% CI = 1.08 – 1.26, and OR = 1.25, 95% CI = 1.16 – 1.34, respectively) and recessive models (OR = 1.44, 95% CI = 1.18 – 1.77, and OR = 1.49; 95% CI = 1.23 – 1.80, respectively). Thus, rs3757328, rs6940552, and rs9261204 in *ZNRD1-AS1* are all associated with increased some cancer risk in an Asian population.

## INTRODUCTION

Long noncoding RNAs (lncRNAs) are a class of RNAs greater than 200 nucleotides in length that are not translated into proteins [1]. The expression of lncRNAs is cell type- and tissue-dependent, which distinguishes them from protein-coding genes [2]. The secondary structures of the lncRNAs can dictate their functions in various cellular processes and diseases [3]. Some lncRNAs activate the oncogenic signaling pathways to drive cancer phenotypes [4]. For example, lncRNA *HULC* promotes the epithelial-to-mesenchymal transition phenotype and tumorigenesis in both pancreatic and gastric cancer cells [5, 6].

Single nucleotide polymorphisms (SNPs) in lncRNAs can also promote cancer development and progression. For example, the TT genotype of rs12826786 in *HOTAIR* was found to increase breast cancer susceptibility [7]. Expression quantitative trait loci (eQTLs) in the lncRNA *CARD8* are susceptibility markers for cervical cancer [8]. The C/T genotype of rs3787016 in the lncRNA *POLR2E* was associated with a decreased risk of esophageal squamous cell carcinoma [9]. Collectively, these data indicate SNPs in lncRNAs have important roles in tumorigenesis and as prognostic biomarkers.

Human zinc ribbon domain containing 1 (*ZNRD1*) is involved in the development of multiple cancers [10]. Interestingly, three SNPs in the lncRNA *ZNRD1-AS1* (rs3757328, rs6940552, and rs9261204), which lies in the upstream region of the *ZNRD1* gene, were found to inhibit *ZNRD1* expression and decrease the risk of cervical cancer [11]. However, several studies have demonstrated that eQTLs in *ZNRD1-AS1* increased the risk of hepatocellular carcinoma (HCC) [12, 13] and lung cancer [10]. These conflicting results for rs3757328, rs6940552, and rs9261204 in *ZNRD1-AS1* need to be further studied. Therefore, we investigated the effects of rs3757328, rs6940552, and rs9261204 in *ZNRD1-AS1* on cancer susceptibility in this study.

## RESULTS

### Study characteristics

Four case-control articles [10–13], which included 5,293 cases and 5,440 controls, were included in our meta-analysis. All of the eligible studies were comprised of Asian populations. In one study, Li et al, investigated both lung cancer and bladder cancer. We therefore considered this study as two independent studies in our analysis (Table 1). The studies were all published between June 15, 2015 and June 30, 2016. The sample size range was 1,000 to 3,067. Finally, the studies investigated distinct tumor types (HCC, lung cancer, bladder cancer, and cervical cancer, Table 1).

**Table 1:** Characteristics of studies on the association between SNPs in *ZNRD1-AS1* and cancer

Author	Year	Ethnicity	Cases	Controls	Type of cancer	Single Nucleotide Polymorphisms	Genotyping Method	Quality Score
						*rs3757328*		
Liu	2016	Chinese	1507	1560	HCC	*rs6940552*	TaqMan	8
						*rs9261204*		
						*rs3757328*		
Li	2016	Chinese	500	500	lung cancer	*rs6940552*	PCR-RFLP	6
						*rs9261204*		
						*rs3757328*		
Li	2016	Chinese	500	500	bladder cancer	*rs6940552*	PCR-RFLP	6
						*rs9261204*		
						*rs3757328*	Sequenom	
Guo	2015	Chinese	1486	1536	cervical cancer	*rs6940552*	MassARRAY	8
						*rs9261204*	iPLEX platform	
						*rs3757328*	Sequenom	
Wen	2015	Chinese	1300	1344	HCC	*rs6940552*	MassARRAY	8
						*rs9261204*	iPLEX platform	

Abbreviations: HCC, hepatocellular carcinoma; PCR-RFLP, polymerase chain reaction-restriction fragment length polymorphism.

All studies explored the relationships between SNPs in *ZNRD1-AS1* (rs3757328, rs6940552, and rs9261204) and cancer risk. The genotyping methods included TaqMan in one study, PCR-restriction fragment length polymorphism in two studies, and the Sequenom MassARRAY iPLEX platform in two studies (Table 1). The methodological quality of each study was evaluated using the Newcastle-Ottawa Scale (NOS). All the studies scored at least 6 on this scale (Supplemental Table S1).

The genotypes for rs3757328, rs6940552, and rs9261204 included AA, GA, and GG. Hardy-Weinberg equilibrium test statistics indicated that the probability of the null hypothesis for most of the genotypes was correct (Table 2).

**Table 2:** Genotype distributions of rs3757328, rs6940552 and rs9261204 in *ZNRD1-AS1*

Year	Author	Case			Control			*P* for HWE in controls
***rs3757328***		**GG**	**GA**	**AA**	**GG**	**GA**	**AA**	
2016	Liu	1038	362	43	1146	375	33	0.72
2016	Li	305	175	20	340	150	10	0.16
2016	Li	337	146	17	340	150	10	0.16
2015	Guo	946	363	35	950	435	51	0.89
2015	Wen	920	319	40	976	333	18	0.08
**rs6940552**		**GG**	**GA**	**AA**	**GG**	**GA**	**AA**	
2016	Liu	958	461	73	1048	453	52	0.72
2016	Li	265	179	56	289	169	42	0.02
2016	Li	274	175	51	289	169	42	0.02
2015	Guo	872	413	61	867	495	86	0.17
2015	Wen	819	404	58	884	402	36	0.22
***rs9261204***		**AA**	**GA**	**GG**	**AA**	**GA**	**GG**	
2016	Liu	853	521	88	976	510	67	0.97
2016	Li	228	207	65	280	180	40	0.15
2016	Li	241	200	59	280	180	40	0.15
2015	Guo	821	438	57	819	521	91	0.51
2015	Wen	741	449	64	836	432	50	0.53

Abbreviations: HWE, Hardy-Weinberg equilibrium.

### Quantitative synthesis

Overall, no significant association between rs3757328 and cancer risk was observed in any of the models tested (heterozygous model: odds ratio [OR] = 1.013; 95% confidence interval [CI] = 0.887 – 1.158; *P* = 0.07 for the heterogeneity test, I^2^ = 53.9%; homozygous model: OR = 1.489; 95% CI = 0.905 – 2.449; *P* = 0.005 for the heterogeneity test, I^2^ = 72.9%; dominant model: OR = 1.050; 95% CI = 0.898 – 1.228, P = 0.012 for the heterogeneity test, I^2^ = 68.9%; recessive model: OR = 1.474; 95% CI = 0.925 – 2.342, *P* = 0.011 for the heterogeneity test, I^2^ = 69.2%; additive: OR = 1.077; 95% CI = 0.921 – 1.260, *P* = 0.002 for the heterogeneity test, I^2^ = 76.1%, Table 3).

**Table 3:** ORs and 95% CI for cancers and *ZNRD1-AS1* rs3757328, rs6940552 and rs9261204 under different genetic models

Genetic models	n	OR (95% CI)	*P* (OR)	Model(method)	I-square(%)	*P* (H)	*P* (Begg)	*P* (Egger)
***rs3757328***								
Heterozygous model (GA *vs* GG)	5	1.013 (0.887, 1.158)	0.844	R(D-L)	53.9	0.070	0.806	0.355
Homozygous model (AA *vs* GG)	5	1.489 (0.905, 2.449)	0.117	R(D-L)	72.9	0.005	0.462	0.240
Dominant model (GA+AA *vs* GG)	5	1.050 (0.898, 1.228)	0.541	R(D-L)	68.9	0.012	0.806	0.351
Recessive model (AA *vs* GA+GG)	5	1.474 (0.925, 2.342)	0.103	R(D-L)	69.2	0.011	0.462	0.260
Additive (A *vs* G)	5	1.077 (0.921, 1.260)	0.352	R(D-L)	76.1	0.002	0.462	0.340
***rs6940552***								
Heterozygous model (GA *vs* GG)	5	1.035 (0.907, 1.180)	0.611	R(D-L)	56.2	0.058	0.806	0.550
Homozygous model (AA *vs* GG)	5	1.271 (0.902, 1.790)	0.170	R(D-L)	72.9	0.005	1.000	0.237
Dominant model (GA+AA *vs* GG)	5	1.070 (0.911, 1.256)	0.411	R(D-L)	73.4	0.005	0.806	0.537
Recessive model (AA *vs* GA+GG)	5	1.248 (0.925, 1.684)	0.147	R(D-L)	65.8	0.020	0.806	0.221
Additive (A *vs* G)	5	1.085 (0.830, 1.266)	0.298	R(D-L)	80.3	<0.001	0.462	0.513
***rs9261204***								
Heterozygous model (GA *vs* AA)	5	1.141 (0.956, 1.362)	0.145	R(D-L)	76.6	0.002	0.221	0.271
Homozygous model (GG *vs* AA)	5	1.346 (0.889, 2.038)	0.161	R(D-L)	83.1	<0.001	0.462	0.250
Dominant model (GA+GG *vs* AA)	5	1.180 (0.953, 1.461)	0.129	R(D-L)	85.5	<0.001	0.221	0.243
Recessive model (GG *vs* GA+AA)	5	1.266 (0.897, 1.788)	0.180	R(D-L)	76.6	0.002	0.462	0.307
Additive (G *vs* A)	5	1.164 (0.955, 1.418)	0.132	R(D-L)	88.8	<0.001	0.462	0.250

Abbreviations: OR, Odds ratio; CI, confidence intervals; *P* (H), *P* for heterogeneity; n, number of included studies; R, random-effects model; D-L, DerSimonian-Laird method.

**Figure 1:**
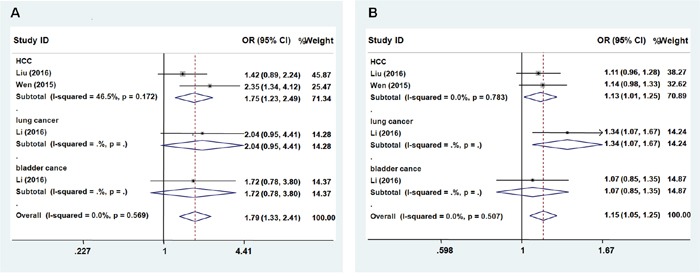
Forest plot of cancer risk associated with *ZNRD1-AS1* polymorphism rs3757328 Models represented in **A**. recessive and **B**. allele contrast.

We next evaluated the effect of the rs3757328 polymorphism on the risk of cancer among the subgroups by cancer type. We observed the rs3757328 in *ZNRD1-AS1* was significantly associated with an increased risk of some cancer types (HCC, lung cancer, and bladder cancer) except cervical cancer (occurs only in women) both in the recessive model (Figure 1A, Recessive model: OR = 1.79; 95% CI = 1.33 – 2.41, *P* = 0.569 for the heterogeneity test, I^2^ = 0.0%) and additive genetic model (Figure 1B, Additive: OR = 1.15; 95% CI = 1.05–1.25, *P* = 0.507 for the heterogeneity test, I^2^ = 0.0%). The A allele of rs3757328 was significantly associated with an increased risk of some cancers compared with G allele.

**Figure 2:**
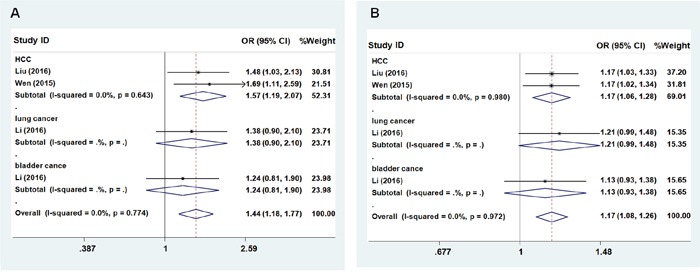
Forest plot of cancer risk associated with *ZNRD1-AS1* polymorphism rs6940552 Models represented in **A**. recessive and **B**. allele contrast.

Similar to the results for rs3757328, no significant association was observed between rs6940552 in *ZNRD1-AS1* and cancer risk (heterozygous model: OR = 1.035; 95% CI = 0.907 – 1.180; *P* = 0.058 for the heterogeneity test, I^2^ = 56.2%; homozygous model: OR = 1.271; 95% CI = 0.902 – 1.790; *P* = 0.005 for the heterogeneity test, I^2^ = 72.9%; dominant model: OR = 1.070; 95% CI = 0.911 – 1.256, P = 0.005 for the heterogeneity test, I^2^ = 73.4%; recessive model: OR = 1.248; 95% CI = 0.925 – 1.684; *P* = 0.020 for the heterogeneity test, I^2^ = 65.8%; additive: OR = 1.085; 95% CI = 0.830 – 1.266, *P* < 0.001 for the heterogeneity test, I^2^ = 80.3%, Table 3). We next evaluated the effects among the subgroups, and found that the A allele of rs6940552 significantly increased cancer risk (HCC, lung cancer, and bladder cancer) except cervical cancer (Figures 2A and 2B, recessive model: OR = 1.44; 95% CI = 1.18–1.77, *P* = 0.774 for the heterogeneity test, I^2^ = 0.0%; additive: OR = 1.17; 95% CI = 1.08–1.26, *P* = 0.972 for the heterogeneity test, I^2^ = 0.0%).

**Figure 3:**
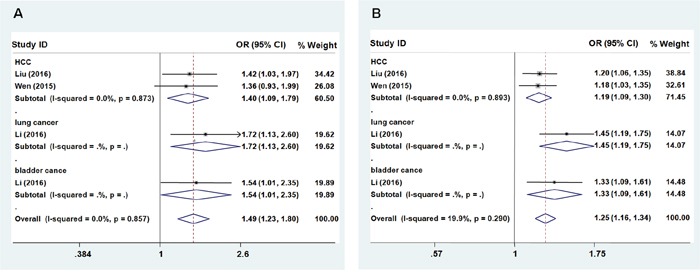
Forest plot of cancer risk associated with *ZNRD1-AS1* polymorphism rs9261204 Models represented in **A**. recessive and **B**. allele contrast.

No significant association between rs9261204 in *ZNRD1-AS1* and cancer risk was found (heterozygous model: OR = 1.141; 95% CI = 0.956 – 1.362; *P* = 0.002 for the heterogeneity test, I^2^ = 76.6%; homozygous model: OR = 1.346; 95% CI = 0.889 – 2.038; *P* < 0.001 for the heterogeneity test, I^2^ = 83.1%; dominant model: OR = 1.180; 95% CI = 0.953 – 1.461, P < 0.001 for the heterogeneity test, I^2^ = 85.5%; recessive model: OR = 1.266; 95% CI = 0.897 – 1.788, *P* = 0.002 for the heterogeneity test, I^2^ = 76.6%; additive: OR = 1.164; 95% CI = 0.955 – 1.418, *P* < 0.001 for the heterogeneity test, I^2^ = 88.8%, Table 3). Further subgroup analyses showed that the G allele of rs9261204 significantly increased cancer risk(HCC, lung cancer, and bladder cancer) except cervical cancer (Figures 3A and 3B, recessive model: OR = 1.49; 95% CI = 1.23–1.80, *P* = 0.857 for the heterogeneity test, I^2^ = 0.0%; additive: OR = 1.25; 95% CI = 1.16–1.34, *P* = 0.290 for the heterogeneity test, I^2^ = 19.9%).

We also performed sensitivity analyses through removal of the individual study one by one. The results indicated that the study by Guo et al. [11] predominantly contributed to the observed heterogeneity in all models. Removal of this study significantly reduced the heterogeneity. These results showed that the study by Guo et al. focused on cervical cancer markly changed the pooled OR.

### Publication bias

Potential publication biases was evaluated using the Egger’s and Begg’s tests. The results provided statistical evidence for the absence of publication bias in all models (Table 3).

## DISCUSSION

SNPs in lncRNAs contribute to the development of various cancers [7, 8, 14]. In this study, we analyzed the associations between three SNPs (rs3757328, rs6940552, and rs9261204) in *ZNRD1-AS1* and cancer risk. Our data indicated that these SNPs are correlated with an increased risk of several cancers in an Asian population.

The *ZNRD1* protein contains two zinc ribbon domains [15]. It catalyzes the transcription of DNA into RNA and is a potential therapeutic target for various diseases [16, 17]. Reduced *ZNRD1* expression was observed in human gastric cancer [18, 19]. Interestingly, *ZNRD1* was found to suppress CDK4, Cyclin D1, and p21 [20] and inhibit the growth of gastric cancer and leukemia cells in vitro [17, 21]. Previous studies have indicated that *ZNRD1-AS1* contribute to tumorigenesis through negative regulation of the *ZNRD1* gene [10]. EQTLs analysis has demonstrated that SNPs in *ZNRD1-AS1* regulate of *ZNRD1* expression [22, 23].

Three SNPs in *ZNRD1-AS1* (rs3757328, rs6940552 and rs9261204) have been associated with an increased risk of several cancers. Wen et al. [12] reported three of the SNPs (rs3757328, rs7769930, and rs694055) were associated with an increased risk of HCC. Li et al. [10] demonstrated that the G allele of rs9261204 increased the risk of lung cancer by 1.45-fold compared to the A allele. Nevertheless, these three SNPs (rs3757328, rs7769930, and rs694055) in *ZNRD1-AS1* decreased the risk of cervical cancer [11]. In this meta-analysis, we evaluated the effect of these three SNPs on the risk of cancer among the subgroups by cancer type. We found that the A allele of SNP rs3757328, A allele of SNP rs6940552, and G allele of SNP rs9261204 in *ZNRD1-AS1* were associated with increased risk of some cancer types (HCC, lung cancer, and bladder cancer) except cervical cancer.

The meta-analysis had limitations. First, only five individual studies (focused on HCC, lung cancer, bladder cancer, and cervical cancer) were included in our analysis, which impacted the quality of our results. Second, our analysis was limited to individuals of Asian descent. Therefore, the effects of the SNPs on non-Asian populations are not yet clear, and further studies are necessary to confirm our results.

In conclusion, our data indicate that three SNPs in *ZNRD1-AS1* were correlated with an increased risk of several cancers. These results must be further evaluated in large-scale, randomized controlled trials involving different ethnic populations and cancers.

## MATERIALS AND METHODS

### Search strategy

We searched the PubMed, Embase, and Web of Science databases for studies performed prior to June 30, 2016 that reported an association between SNPs in *ZNRD1-AS1* and cancer risk. This comprehensive literature search was performed using free-text words combined with Medical Subject Headings, such as “*ZNRD1*”, “*ZNRD1-AS1*”, or “lncRNA” and “cancer”, “carcinoma”, “tumor”, “tumour”, or “neoplasm” and “polymorphism”, “variation”, “variant”, “SNP”, “mutation”, or “genotype”. The references cited in the retrieved articles were also reviewed to identify additional eligible studies (Supplemental Figure S1).

### Inclusion and exclusion criteria

The study inclusion criteria were the following: (1) case-control design; (2) evaluated associations between SNPs in *ZNRD1-AS1* and cancer; (3) provided sufficient data for the allele and genotype frequencies (i.e., rs3757328, rs6940552 or rs9261204); (4) published in English with the full-text article available; and (5) involved human subjects. The exclusion criteria were: (1) review article or commentary; (2) non-English publication; (3) replication of a previous study; and (4) non-human subjects.

### Data extraction

Two investigators (PYW and JHL) independently extracted the data from each study, including the surname of the first author, publication year, type of cancer, numbers of cases and controls, ethnicity, genotype platform, and SNP genotype. Disagreement was resolved through a discussion with a third reviewer (YML).

### Quality assessment

The methodological quality of each eligible study was evaluated using the NOS. Each study was evaluated based on the selection, comparability, and exposure scores. Summary scores ranging from 0 to 9 points were calculated. Higher score were indicative of a lower risk of bias.

### Statistical analysis

Allele contrast, dominant, recessive, homozygous, and heterozygous models were used to analyze the associations between SNPs in *ZNRD1-AS1* and cancer risk. We calculated ORs and 95% CIs in order to estimate the strength of the associations. The significance of the ORs was determined using Z tests. Heterogeneity between studies was assessed using the Chi square-based Q statistic. A random effects (DerSimonian-Laird method) or fixed effect (Mantel-Haenszel method) model was used to calculate pooled effect estimates in the presence (*P* < 0.10) or absence (*P* > 0.10) of heterogeneity, and subgroup analysis by cancer type was further performed. Sensitivity analysis was performed by excluding one study at a time and recalculating the risk effect. Begg’s and Egger’s tests were performed to evaluate publication bias. Data analysis was performed using the Stata software, version 12.0 (Stata Corporation; College Station, TX, USA). A *P* value < 0.05 was considered statistically significant.

## SUPPLEMENTARY FIGURE AND TABLE


