# Supplementary material for: Single nucleotide polymorphisms in ZNRD1-AS1 increase cancer risk in an Asian population
Source: Oncotarget. 2016 Dec 28;8(6):10064–70. doi: 10.18632/oncotarget.14334 (PMC5354641; doi:10.18632/oncotarget.14334)
Supplement: Supplementary file 1 [file oncotarget-08-10064-s001.pdf]

Single nucleotide polymorphisms in *ZNRD1-AS1* increase cancer risk in an Asian population

SUPPLEMENTARY FIGURE AND TABLE

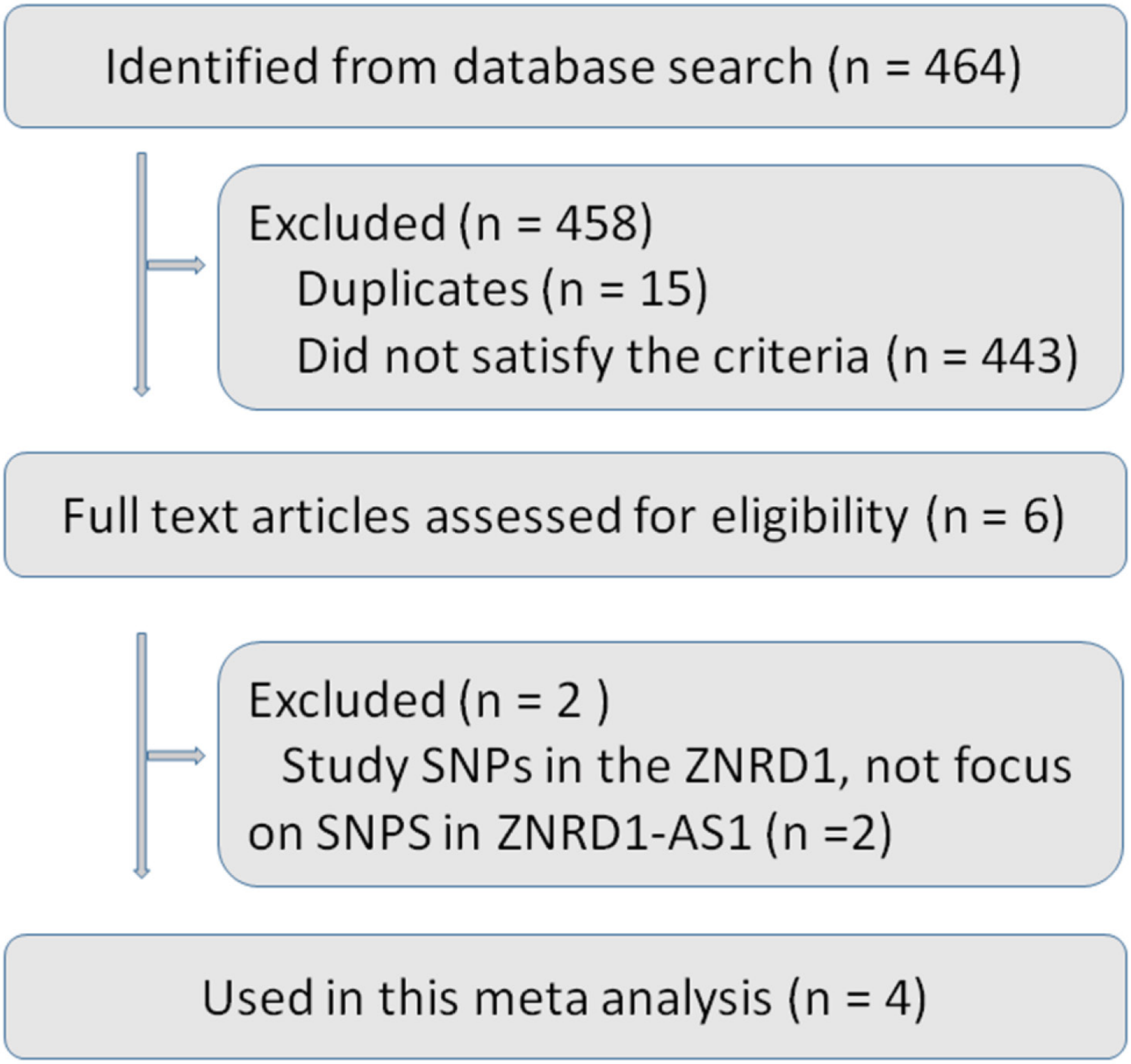

Supplementary Figure 1: Process of study selection.

Supplementary Table 1: Newcastle-Ottawa quality assessment scale for each included study

Studies	Selection				Comparability		Exposure			Total quality score
	Case definition adequate	Representativeness of the cases	Selection of controls	Definition of controls	Adjustment for age and gender	Adjustment for other risk factors	Ascertainment of exposure	Uniform method of ascertainment	Non-response rate	
Liu	1	1	1	1	1	1	1	1	0	8
Li	1	0	1	0	1	1	1	1	0	6
Guo	1	1	1	1	1	1	1	1	0	8
Wen	1	1	1	1	1	1	1	1	0	8
